# Reputation Effects in Public and Private Interactions

**DOI:** 10.1371/journal.pcbi.1004527

**Published:** 2015-11-25

**Authors:** Hisashi Ohtsuki, Yoh Iwasa, Martin A. Nowak

**Affiliations:** 1 Department of Evolutionary Studies of Biosystems, School of Advanced Sciences, SOKENDAI (The Graduate University for Advanced Studies), Hayama, Kanagawa, Japan; 2 Department of Biology, Faculty of Sciences, Kyushu University, Fukuoka, Japan; 3 Program for Evolutionary Dynamics, Harvard University, Cambridge, Massachusetts, United States of America; 4 Department of Mathematics, Harvard University, Cambridge, Massachusetts, United States of America; 5 Department of Organismic and Evolutionary Biology, Harvard University, Cambridge, Massachusetts, United States of America; Philadelphia, UNITED STATES

## Abstract

We study the evolution of cooperation in a model of indirect reciprocity where people interact in public and private situations. Public interactions have a high chance to be observed by others and always affect reputation. Private interactions have a lower chance to be observed and only occasionally affect reputation. We explore all second order social norms and study conditions for evolutionary stability of action rules. We observe the competition between “honest” and “hypocritical” strategies. The former cooperate both in public and in private. The later cooperate in public, where many others are watching, but try to get away with defection in private situations. The hypocritical idea is that in private situations it does not pay-off to cooperate, because there is a good chance that nobody will notice it. We find simple and intuitive conditions for the evolution of honest strategies.

## Author Summary

We study the evolution of cooperation based on reputation. This mechanism is called indirect reciprocity. In a world of binary reputations, people help a good individual but do not help a bad one. They also monitor their own reputation to receive reciprocation from others. We propose a novel model of indirect reciprocity where two types of interactions exist. In a public interaction your behavior is always observed by others. In a private interaction, your behavior is less likely to be observed. We study the competition between honest and hypocritical strategies. The former always help good individuals, whereas the latter do so only in private interactions. We describe conditions for the evolution of honest strategies.

## Introduction

Most human interactions occur in situations where repetition is possible and reputation is at stake. Repeated interactions in a group of players facilitate evolution of cooperation via indirect reciprocity [1, 2]: here players use conditional strategies that depend on what has happened between others. Cooperation is costly but can establish a good reputation. Others might preferentially cooperate with those who have a good reputation. Many studies explore theoretical [3–49] and empirical [50–70] aspects of indirect reciprocity. Experiments reveal that people help those who help others [50, 52–57, 59, 60, 62–67, 70]. Reputation is a strong driving force of prosocial behavior [54, 61, 71–80]. Internet commerce is based to a large extent on reputation systems: buyers are sensitive to sellers’ reputation [76, 81–87].

The standard framework of indirect reciprocity assumes that each interaction consists of a donor and a recipient. The donor can choose between cooperation and defection. If the donor cooperates, her cost is *c* and the benefit for the recipient is *b*. If the donor defects, there is no cost and no benefit. A crucial parameter is the benefit-to-cost ratio, *b*/*c*.

Many studies of indirect reciprocity so far assumed that social interactions are public, which means that everyone is informed about the outcome of these interactions. In reality, however, social information is often incomplete. A previous study [6] showed that if donors remember the reputation of their recipient only with probability *q*, cooperation evolves if *b*/*c* > 1/*q*, suggesting that incomplete information hinders indirect reciprocity.

But in this paper we study another source of incomplete information; the absence of observers. Engelmann & Fischbacher [63] found that donors helped recipients substantially more when their reputation score was seen by others than when it was not publicly announced. Accumulating evidence suggests that humans are very sensitive to cues of being observed, and change their social behavior accordingly [72, 73, 75, 77, 79]. These facts motivate us to study strategies in indirect reciprocity when two types of interactions differing in observability are mixed. In public interactions the donor’s action always affects his reputation, but in private ones it affects his reputation only with probability *q* and otherwise his reputation is unchanged. In our framework people can use different strategies depending on whether they are in private or in public situations. Moreover, observers can evaluate private and public interactions with different assessment rules. For example, they could be indifferent to displays of public cooperation, but very much reward private cooperation, if they hear about it, or vice versa. Crucial questions such as those have not yet been studied in the context of indirect reciprocity.

## Models

We have constructed a theoretical model of indirect reciprocity to study the effect of the mixture of public and private interactions. We consider an infinitely large population of players who are engaged in indirect reciprocity interactions. In each time step, they are randomly matched to form a pair that consists of a donor and a recipient. The donor can choose between cooperation and defection. If the donor cooperates, her cost is *c* and the benefit for the recipient is *b*. If the donor defects, there is no cost and no benefit.

In addition, each interaction within a pair is public with probability, *p*, and private with probability 1 − *p*. For simplicity we assume that a public interaction is always observed, while a private interaction is observed only with probability *q*. Therefore, on average, an interaction is observed with probability, $\bar{q}=p+\left(1-p\right)q$.

**Fig 1:**
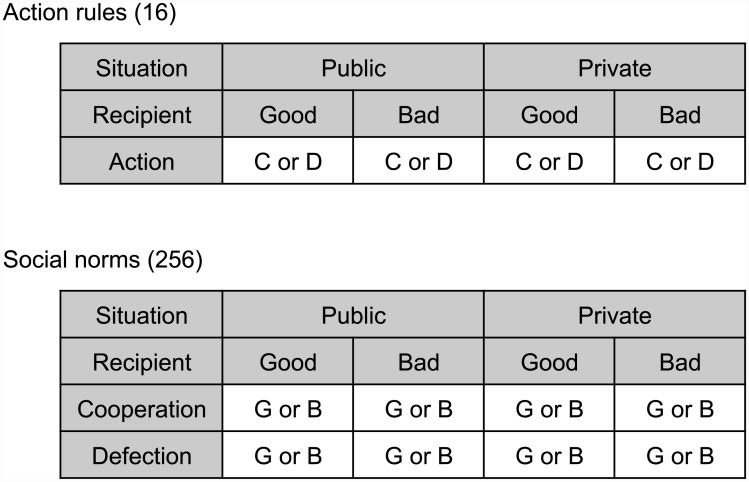
An action rule specifies how a donor behaves in ‘public’ and ‘private’ interactions with recipients who have either a ‘good’ or ‘bad’ reputation. The donor’s choice is binary: cooperate or defect. There are 16 different action rules. A social norm describes how (potential) observers evaluate an interaction. The donor’s reputation is updated dependent on the type of interaction (public or private), the recipient’s reputation (good or bad) and the donor’s action (cooperate or defect). There are 256 such second-order social norms.

We consider a world with binary reputation scores: each player has either a good or a bad reputation, depending on his previous actions. We assume that everyone knows and agrees on others’ reputation scores. A strategy in indirect reciprocity games is called an action rule. We have 16 different action rules. Each action rule specifies whether to cooperate or to defect given that the situation is either public or private and given that the reputation of the recipient is either good or bad (Fig 1). For example, the action rule CDCD (honest) means: cooperate with good recipients and defect with bad ones, no matter whether the situation is public or private. In contrast, CDDD (hypocrite) means: in public situations the actor will cooperate with good recipients and defect with bad ones, but in private situations the actor will always defect. There is also unconditional cooperation, CCCC, and unconditional defection, DDDD.

If the other people in the population are informed about an interaction, then they update the reputation of the actor by using their social norm. We consider second order social norms [14, 15], which depend on the action of the donor, the reputation of the recipient and whether the interaction was public or private. Thus, there are 256 possible social norms (Fig 1). We assume that all people in the population use the same social norm. After a single game interaction, players leave the pair and go back to the population pool to wait for another recruitment. After a sufficiently large number of interactions, natural selection acts on action rules according to their payoffs. A goal of our analysis is to identify which of the 16 strategies (action rules) are evolutionarily stable for each of the 256 norms.

Because of the assumption of an infinitely large population, no two players can ever meet more than once, so the chance of any forms of directly reciprocity is excluded from the model. We further assume that the time scale of natural selection is much slower than that of social interactions and reputation updates. Throughout the analysis, therefore, we can assume that the fraction of time that one has a good reputation is at an equilibrium level. The model presented here can further be extended by incorporating the effect of errors and finite time horizon. Details of the present model as well as those extensions are described in S1 Text.

Our analysis uses the method of dynamic programming [18, 27]. Details are provided in S1 Text, but to grasp the idea, here we show one example. Consider the social norm that regards cooperation as good and defection as bad irrespective of the other factors. Such a norm is called “Scoring” [11, 15]. We ask, for example, if the action rule CDCD (honest) is an evolutionarily stable strategy (ESS) under this norm. For that purpose, we temporarily assume that everyone adopts CDCD.

Dynamic programming allows us to calculate the value of a good reputation, *v*, which reflects the advantage of possessing a good reputation over a bad one. In our example it is calculated as $v=b/\bar{q}>0$ (see S1 Text for the derivation), suggesting that keeping a good reputation is advantageous, but it is qualitatively a trivial consequence of everyone using CDCD.

With the value *v*, we check if each digit of the action rule CDCD follows the optimal behavior, because otherwise it is not an ESS. In our example, cooperation in public interactions costs *c* to the donor but makes his reputation good, by which he obtains the future benefit, *v*. Therefore, the relative size of *c* and *v* matters. If *v* < *c* then cooperation in public interactions does not pay, so the first digit of CDCD is not optimal. If *v* > *c* then defection in public interactions does not pay, so the second digit of CDCD is not optimal. In either way, CDCD does not follow the optimal behavior, so it is not ESS. An analysis of this kind is systematically repeated for each of the 256 social norms and for each of the 16 action to find all ESS.

## Results

**Fig 2:**
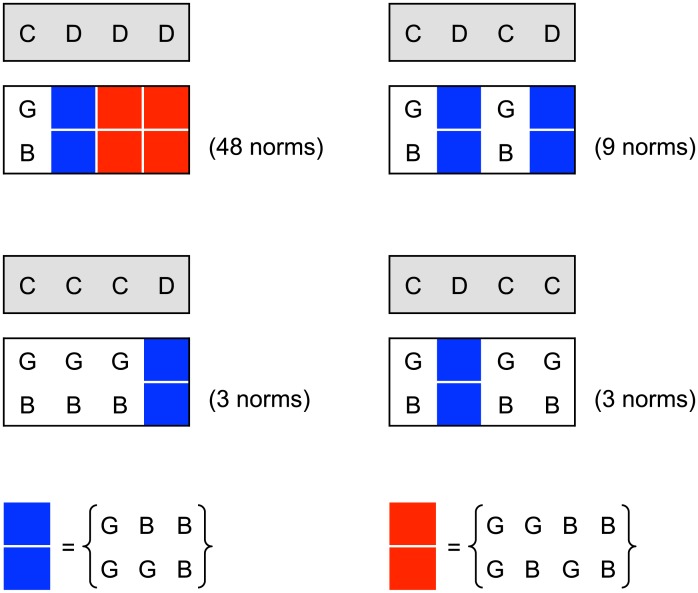
There are 9 social norms that make CDCD evolutionarily stable, 3 social norms that stabilize CCCD, and 3 social norms that stabilize CDCC. There are 48 social norms that make CDDD evolutionarily stable. Note that CDDD only leads to partial cooperation. The other three action rules achieve perfect cooperation except CDCD when used with the social norm where both wild cards are BB. The hypocrite strategy CDDD is robust for a wider range of social norms, but the honest strategy CDCD achieves higher levels of cooperation.

We find that notorious DDDD is always ESS. The question is which other strategies are ESS and for what conditions. We note that DDDD is ESS for any social norm, while all cooperative strategies require specific social norms to be ESS. In Fig 2, we show social norms that allow CDDD (hypocrite) and CDCD (honest), respectively, to be ESS.

**Fig 3:**
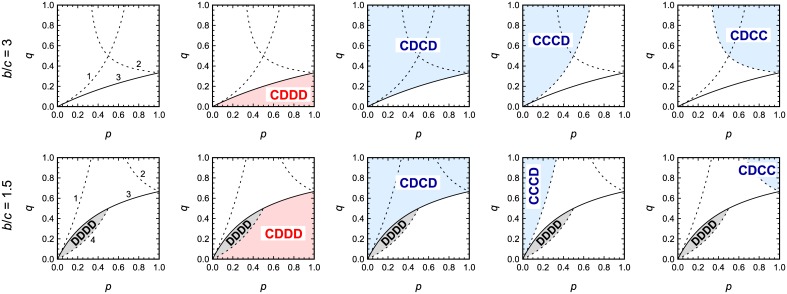
Analytical classification of indirect reciprocity with public and private interactions. Parameter regions for the most cooperative, evolutionarily stable action rules are shown. *p* denotes the frequency of public interactions; *q* denotes the probability that a private interaction is observed. Perfect cooperation (using CDCD) is realized above the solid line labeled as ‘3’, whereas only partial cooperation (using CDDD) is achieved below. There are also parameter regions where the lenient strategies, CCCD and CDCC, are ESS. For *b*/*c* < 2 there exists a parameter region where no ESS other than DDDD is found (shown in gray). The curves are given by 1: *q* = *p*/[(*r* − 1)(1 − *p*)], 2: *q* = *p*/[(*r* + 1)*p* − 1], 3: *q* = *p*/(*p* + *r* − 1), and 4: *q* = (*r* − 1)*p*/(1 − *p*), where *r* ≡ *b*/*c*.

We obtain the following results. If *b*/*c* is less than both $\bar{q}/q$ and $\bar{q}/p$ then the only ESS is DDDD. If $b/c>\bar{q}/p$ then CDDD is ESS. CDDD achieves only partial cooperation. If $b/c>\bar{q}/q$ then CDCD is ESS; this is the crucial condition for the evolution of an honest strategy. Social norms that support the evolutionary stability of CDCD turn out to be the combinations of “Simple-Standing”, “Kandori” (also known as “Stern-Judging”), and “Shunning” social norms [15, 19, 21] (see Fig 2). There is also the possibility that more lenient strategies can evolve if stronger conditions are fulfilled. If $b/c>\bar{q}/\left[\left(1-p\right)q\right]$ then CCCD is ESS. If $b/c>\bar{q}/\left(pq\right)$ then CDCC is ESS. All three strategies, CDCD, CCCD and CDCC, achieve full cooperation for appropriate social norms. S1 Text describes all the ESS we found in our analysis. Note that our model allows multiple ESS. In Fig 3, we show ESS that achieve the highest level of cooperation for the given parameters, because either group competition [17, 19, 22, 30] or intergroup contingent movement by players [27] is likely to favor such ESS. Fig 3 also distinguishes between the two cases, *b*/*c* > 2 and 1 < *b*/*c* < 2. Quite interestingly, in the latter case we find a pocket inside our parameter space (shown in gray in Fig 3) where no combination of *p* and *q* allows the evolutionary stability of action rules other than DDDD. This result is an unexpected consequence of the interplay between public and private interactions; the reputation that one acquires in a public interaction affects one’s private interactions, and vice versa.

Our results have simple intuitive justifications. Consider the crucial condition, $b/c>\bar{q}/q$, that needs to hold for the honest strategy, CDCD, to be the most cooperative ESS. The most dangerous invader is CDDD. Let us now examine the situation where you have a good reputation and meet another person with a good reputation in a private situation. If you cooperate then you lose *c*, but maintain a good reputation. If you defect then you save *c*, but obtain a bad reputation with probability *q*. Once it occurs you will not receive cooperation (losing *b*) until you recover a good reputation. To regain a good reputation your help must be observed by a third party, which occurs with probability $\bar{q}$ per interaction. Therefore you must wait $1/\bar{q}$ rounds. Thus, cooperation costs *c*, whereas defection costs $qb/\bar{q}$. Cooperation is less costly if $c<qb/\bar{q}$ yielding the desired condition. Similar intuitions exist for other conditions and are described in S1 Text.

When comparing with previous models of incomplete information, one may wonder why the evolutionary condition of the honest strategy, CDCD, is now more relaxed. Note that $b/c>\bar{q}/q$ requires a lower benefit-to-cost ratio than the previously known condition, *b*/*c* > 1/*q*. The explanation is as follows. In previous models, observers are always present and your reputation as a donor is always updated, but people sometimes fail to remember your reputation. In contrast, our current model assumes that observers are sometimes not present. The absence of observers could be good news for defectors, but not necessarily; once you obtain a bad reputation, it carries over to your future interactions until your next interaction is observed. Therefore, defectors experience more hardship in the current model.

**Fig 4:**
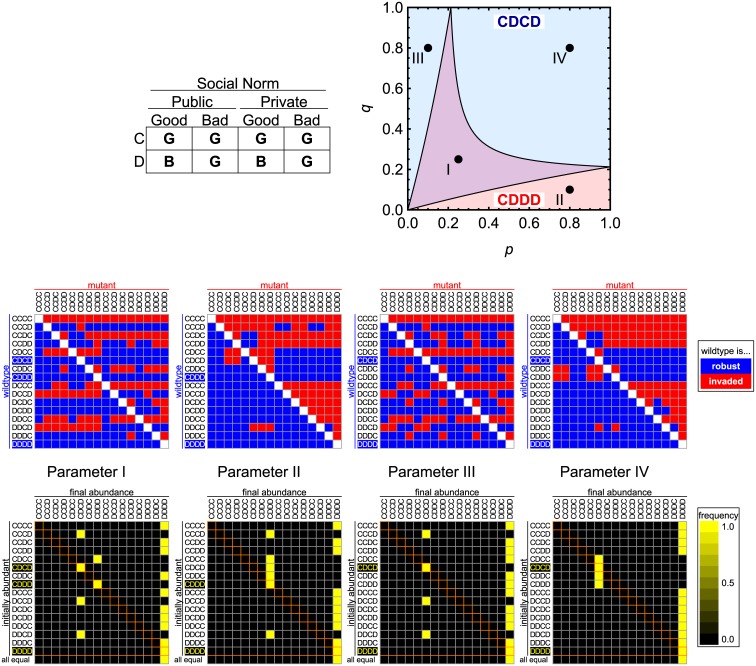
Numerical tests of evolutionary dynamics for a particular social norm which stabilizes both CDCD and CDDD (in addition to DDDD). Top left: the social norm that is studied. Top right: our theoretical prediction of cooperative ESS for various *p* and *q* for *b*/*c* = 5. CDCD is ESS in the blue region. CDDD is ESS in the red region. Both are ESS in the purple region. Middle and Bottom rows: results of deterministic computer simulations. The chance of erroneous reputation assignment is set to *e* = 0.03. Middle row: pairwise comparison between a wildtype (initial frequency = 0.99) and a mutant (= 0.01). Invasion is deemed successful if the mutant’s frequency exceeds 0.01 after a long run. We observe no neutrality. Bottom row: competition among 16 action rules, where one of them is initially abundant (= 0.99) and the others are rare (= 0.01/15 each), or all 16 are initially equally abundant (= 1/16; ‘all equal’ treatment). Our theoretical prediction is in agreement with this numerical analysis. Parameter values (*p*, *q*) are I:(0.25, 0.25), II:(0.8, 0.1), III:(0.1, 0.8) and IV:(0.8, 0.8).

In Fig 4 we compare our analytical results with numerical simulations of evolutionary dynamics. We examine a particular social norm where defection against a ‘good’ recipient leads to bad reputation in public and private situations (when they are observed), while other actions lead to good reputation. The pairwise invasion plots confirm our evolutionary stability analysis for the various parameter regions. We also study convergence to equilibrium points starting from various initial conditions. The system behaves in accordance with our analytical results. Additional numerical tests are performed in S1 Text.

## Discussion

We have studied indirect reciprocity in public and private situations. Our system has three parameters: the benefit-to-cost ratio, *b*/*c*, the frequency of public interactions, *p*, and the probability that private interactions are revealed, *q*. Of fundamental interest is the competition between the honest strategy, CDCD, which cooperates with deserving recipients both in public and private and the hypocritical strategy, CDDD, which only cooperates in public but never in private. We find that for reasonably small *q* values CDCD can prevail over CDDD. The critical *q* for CDCD to be the most cooperative ESS is a declining function of *b*/*c* and an increasing function of *p*. Specifically, we can derive the following prediction: helping in a private interaction is suppressed (i) if the observability *q* is low AND if (ii) private interactions are rare (large *p*); see Fig 3. Empirical studies are needed that examine a mixture of public and private interactions in a laboratory setting or field study to test this prediction.

One is tempted to associate the two key strategies with different motives: CDCD behaves ‘properly’ irrespective of the probability of being observed, while CDDD cooperates (with deserving recipients) only when there is certainty that others will notice the good deed; in contrast it tries to get away with defection when no one is watching. Thus CDCD seems to have ‘higher morals’, while CDDD represents a more utilitarian approach. There is, however, also a cynical interpretation of CDCD: as we have noted this strategy is only stable if the probability to be observed in private interactions is sufficiently high; therefore a CDCD player cooperates in private situations, because on average it does pay-off to do so. Our theoretical results suggest that strategic reputation building [15, 57, 63] is a strong driving force for the evolution of action rules in indirect reciprocity.

## Supporting Information

S1 Text Full analysis of the model. The supporting information is structured as follows. Section 1 describes our full model with maximal generality. Section 2 describes the analytical framework to analyze this full model. Section 3 shows the results of our ESS search. Section 4 explains a reduced model, which directly corresponds to the model described in the main text. Section 5 describes numerical simulations.
(PDF)
