# Supplementary material for: Reputation Effects in Public and Private Interactions
Source: PLoS Comput Biol. 2015 Nov 25;11(11):e1004527. doi: 10.1371/journal.pcbi.1004527 (PMC4659694; doi:10.1371/journal.pcbi.1004527)

Supporting Information (S1 Text):

Reputation effects in public and private interactions

Hisashi Ohtsuki[1], Yoh Iwasa[2] & Martin A. Nowak[3,4,5]

[1]Department of Evolutionary Studies of Biosystems, School of Advanced Sciences, SOKENDAI (The Graduate University for Advanced Studies), Hayama, Kanagawa 240-0193, Japan

[2]Department of Biology, Faculty of Sciences, Kyushu University, Fukuoka 812-8581, Japan

[3]Program for Evolutionary Dynamics, [4]Department of Mathematics, [5]Department of Organismic and Evolutionary Biology, Harvard University, Cambridge MA 02138, USA

Full analysis of the model

1 Model Description

1.1 Indirect reciprocity game

We consider an infinitely large population, where individuals (hereafter called *players*) are engaged in *indirect reciprocity game* (details explained later). Each player is endowed with a binary reputation; either *good* (G) or *bad* (B). We assume that everyone agrees on one's reputation (*i.e.* no two individuals disagree upon the reputation of the same player).

We consider a continuous time model. In each infinitesimal time interval, Δt, a fraction $2\Delta t$ of players are randomly sampled from the population, half as a donor and half as a recipient of indirect reciprocity game. Each donor is paired with a different recipient. Each donor-recipient pair finds themselves in one of the m *situations*, named $S_1, \ldots, S_m$, with non-zero probabilities, $p_1, \cdots, p_m$, respectively. Those situations differ in observability, that is, the probability that the donor's action is observed by others. We assume that donors and recipients are aware of their situation (=how likely they are observed). Table A lists up the symbols used in this Supporting Information.

Each player has an *action rule* that prescribes how to behave in a social interaction. In each indirect reciprocity game, a donor chooses one 'action', either *cooperation* (C) or *defection* (D), with taking into account recipient's reputation and the current situation. Cooperation means that the

Table A: List of symbols

Symbol	Use
a	Action rule
a^*	Best response action rule
b	Benefit of help
c	Cost of help
D_i	Differential in payoffs between good and bad players in situation i
e_i	Reputation-assignment error in situation i
$\hat{e}_i$	$= 1 - 2e_i$
$F_{i,\mathrm{G}}$	Probability of receiving a good reputation when playing with a good recipient in situation i
$F_{i,\mathrm{B}}$	Probability of receiving a good reputation when playing with a bad recipient in situation i
g^*	Equilibrium fraction of good players
$g(t)$	Fraction of good players in the population at time t
i	Index for situations (= 1 (public), = 2 (private))
m	Number of situations (= 2 in most of our analysis)
n	Social norm
p_i	Frequency of situation i
q_i	Observability in situation i
$\bar{q}$	Average observability ($= p_1q_1 + p_2q_2$)
v	Marginal value of a good reputation
ω	Probability that a player proceeds to a next interaction

donor pays the cost $c(> 0)$ to give the benefit $b(> 0)$ to the recipient. Defection means that the donor pays nothing and the recipient gets nothing. We do not consider execution errors (*i.e.* an intended action is always correctly performed).

In situation S_i, the action of the donor in an indirect reciprocity game is observed by a third party (hereafter called *reporter*) with probability q_i. If the action is observed, the reporter refers to the *social norm* in the population, and privately assigns a reputation to the donor. In this process, the reporter mistakenly assigns the opposite reputation to the donor (*i.e.* 'Good' to those who should be labelled 'Bad' according to the social norm, and vice versa). This error occurs with probability $e_i(\leq 1/2)$ in situation S_i. The reporter then tells the new reputation of the donor to the public and everyone accepts this report. With the remaining probability of $1 - q_i$, the action of the donor is not observed. In this case the donor's reputation is not updated at all; it remains the same as the current one.

After a game interaction, the donor and the recipient independently either remains in or leaves the population with probabilities ω and $1 - \omega$, respectively ($0 < \omega < 1$) (continuous-entry model; [14]). We assume that each time one player leaves the population, another player with the same action rule joins it. We further assume that the initial reputation of this new comer is determined probabilistically. In particular, we assume that this probability is equal to the current frequency of good players in the population. That is, a stranger is likely to be deemed good in a population of many good players, and vice versa.

Payoff-dependent natural selection on action rules occurs, but only so infrequently that the separation of time scales is possible; we assume that the time scale of natural selection is much slower than that of social interactions and reputation updates. Therefore we are always able to assume that the frequency of individuals with a good reputation is at its equilibrium value.

In the following, we will concentrate on the case of $m = 2$ (=there are only two situations). Without loss of generality we assume $q_1(1-2e_1) > q_2(1-2e_2)$ holds (we do not study the degenerate case of their being equal), which characterizes the difference in observability between public and private situations. The quantity $q_i(1-2e_i)$ represents how much one's behavior as a donor influences his/her future reputation (for example, to understand the factor $1 - 2e_i$, imagine the most extreme

error rate, $e_i = 1/2$, where donor's new reputation is determined by a random coin flip; in that case whether a donor cooperates or defects is totally irrelevant to his new reputation, so the factor $1 - 2e_i$ is equal to zero). In the following, we call situations S_1 and S_2, *public* and *private* situations, respectively.

1.2 Social norm

We consider second-order social norms; norms that assign a new reputation to donors by taking into account (i) donor's action (cooperation or defection) and (ii) recipient's reputation (good or bad), as well as the situation of the interaction (public or private). A social norm is described by Table B. Each of the eight pivots can be either good (G) or bad (B), reflecting the evaluation of the corresponding donor. There are $2^8 = 256$ social norms in total.

Table B: Second-order social norm

situation	public		private	
donor's action \ recipient's reputation	good	bad	good	bad
cooperation	G/B	G/B	G/B	G/B
defection	G/B	G/B	G/B	G/B

1.3 Action rule

An action rule prescribes the behavior of a donor (either cooperation (C) or defection (D)) conditioned on the recipient's reputation and the situation of the interaction. An action rule is described by Table C. Each of the four pivots can be either cooperation (C) or defection (D). There are $2^4 = 16$ action rules in total. In the following, we call these action rules CCCC, CCCD, ..., and DDDD in short. For example, the action rule CDDC prescribes (i) cooperation in a public interaction with a good recipient, (ii) defection in a public interaction with a bad recipient, (iii) defection in a private interaction with a good recipient, and (iv) cooperation in a private interaction with a bad recipient.

Table C: Action rule

situation	public		private	
recipient's reputation	good	bad	good	bad
action	C/D	C/D	C/D	C/D

2 Analytical Calculations

2.1 Overview

Goals of this section is to answer the following two questions:

(a) Assume that all players share one of the 256 social norms. Given a social norm, which of the 16 action rules is ESS (Evolutionarily Stable Strategy)?

(b) Which combination of a social norm and an action rule (that is ESS under this norm) achieves the highest level of cooperation at an equilibrium?

In order to answer these questions, we fix a social norm, n, in the following analysis and assume that all players use this social norm. We also fix an action rule, a, to study the property of the population dominated by this action rule.

For a given pair of a social norm and an action rule (n, a), we first derive how much a good reputation is beneficial to a player, compared with a bad one. It is called a 'marginal value' of a good reputation, and it can be analytically derived (Section 2.2). The advantage of deriving such a marginal value is that we are able to translate future benefits through a good reputation to one's immediate benefit, thus it enables us to directly compare a genuine immediate benefit (often arises when defecting with others) with a future benefit (often arises through a good reputation, which in turn was obtained by cooperating with others).

Given a marginal value of a good reputation, our next step is to consider evolutionary stability. In particular, we consider the 'best response' action rule against a, that is, the action rule that earns the largest payoff in a sea of a-players among all 16 possible action rules (Section 2.3). If this best response action rule happens to be the same as a we can conclude that a is an ESS, otherwise a is invaded by another strategy so it is not an ESS. Therefore we are able to achieve the goal (a) above.

Our next question is how much fraction of players are cooperating at such an ESS. We introduce reputation dynamics, which describe the change of frequency of good players in the population, and study the behavior of the system at an equilibrium (Section 2.4). By this we can achieve the goal (b) above.

2.2 Marginal value of a good reputation

A marginal value of a good reputation is defined as the maximum total payoff that the player currently with a good reputation can gain from present to future, minus that of the player currently with a bad reputation [18, 27]. In other words, it measures the difference in payoff consequences due to the difference in reputations at a single census point. Throughout the paper we denote this marginal value by v. This value plays a pivotal role in our analysis.

Let us define the average observability of interactions, $\bar{q} = p_1 q_1 + p_2 q_2$. In order to calculate the marginal value, we need to consider different payoff consequences that good and bad reputation holders face, and how long the effect of a current reputation is carried over to future rounds.

Imagine a player who is sampled as a participant of an indirect reciprocity game. First, with probability $(1/2)\bar{q}$ his role is donor and his reputation is subject to update. Because his current reputation is not used when he plays donor, it yields no immediate payoff consequences to him. Also, because his current reputation is subject to update, it will yield no payoff consequences to him in future indirect reciprocity games. Second, with probability $(1/2) \cdot (1 - \bar{q})$ his role is donor and his reputation is not subject to update, suggesting that the current reputation has no immediate payoff consequences to him but that it is carried over to his next interaction. Third, with probability $(1/2)p_1$ the focal player's role is recipient in a public interaction. As a recipient, his current reputation does matter to his immediate payoff, because others may preferentially cooperate with good/bad-reputation holders. Also, because he is a recipient, his reputation is not updated and carried over to his next interaction. Fourth, with probability $(1/2)p_2$ the focal player plays recipient in a private interaction. His current reputation matters to his immediate payoff consequence for the same reason, and his current reputation is carried over to the next interaction.

Taking those four separate cases into account, we obtain the following recursion on v:

$$v = \underbrace{\frac{1}{2}\bar{q}}_{\text{donor, updated}} (0 + \omega \cdot 0) + \underbrace{\frac{1}{2}(1-\bar{q})}_{\text{donor, no update}} (0 + \omega \cdot v) + \underbrace{\frac{1}{2}p_1}_{\text{recipient, public}} (D_1 + \omega \cdot v) + \underbrace{\frac{1}{2}p_2}_{\text{recipient, private}} (D_2 + \omega \cdot v). \tag{2.1}$$

Each term in (2.1) corresponds to each of the four scenarios described above. Inside brackets are the differentials in the immediate payoff between good/bad-reputation holders, plus the effect of a current reputation on future payoffs, which is discounted by ω. Values D_1 and D_2 are not yet known (derived below), but they respectively represent the relative advantage of having a good reputation (compared to having a bad one) as a recipient in a public (D_1) or private (D_2) interaction.

Equation (2.1) leads to

$$v = \frac{p_1 D_1 + p_2 D_2}{2 - (2 - \bar{q})\omega}. \tag{2.2}$$

Note that D_1 and D_2 depend on the action rule adopted in the population, a. For example, if the action rule adopted in the population is a = CDDC, a good player in a public interaction receives cooperation (benefit, b) whereas a bad one does not (benefit, 0), leading to the differential, $D_1 = b - 0 = b$. In contrast, a good individual does not receive cooperation whereas a bad one does in a private interaction, leading $D_2 = 0 - b = -b$. Values of D_1 and D_2 for each of the 16 action rules adopted by resident individuals in the population are summarized in Table D.

2.3 Best response action rule

Once the marginal value of a good reputation, v, is known, we can derive the 'best response' action rule, a^*, (=the action rule that earns the largest payoff) in a population adopting social norm n and dominated by action rule a. The advantage of deriving the best action rule, a^*, is that we can easily check whether action rule a is evolutionarily stable or not. More precisely speaking, a is an ESS if and only if $a^* = a$ (we do not consider the case of multiple best response action rules).

Derivation of the best response action rule can be done quite systematically. For example, let us consider the case where one plays donor and meets a good player in a public interaction, and let us study which action is the more beneficial to the donor, cooperation or defection. The best action depends on what the corresponding column of the social norm (in this case, the 'public' & 'good'

Table D: D_1 and D_2 for each action rule

action rule a	D_1	D_2	$\{2-(2-\bar{q})\omega\}v$
CCCC	0	0	0
CCCD	0	b	$p_2 b$
CCDC	0	$-b$	$-p_2 b$
CCDD	0	0	0
CDCC	b	0	$p_1 b$
CDCD	b	b	b
CDDC	b	$-b$	$(p_1-p_2)b$
CDDD	b	0	$p_1 b$
DCCC	$-b$	0	$-p_1 b$
DCCD	$-b$	b	$(-p_1+p_2)b$
DCDC	$-b$	$-b$	$-b$
DCDD	$-b$	0	$-p_1 b$
DDCC	0	0	0
DDCD	0	b	$p_2 b$
DDDC	0	$-b$	$-p_2 b$
DDDD	0	0	0

column) says (see Table B to observe four columns there), because this column is used to evaluate the donor's behavior.

Suppose that the corresponding column says 'G-B', meaning that cooperation gives the donor a good reputation while defection gives him a bad reputation in a public interaction with a good player. If the donor cooperates, he immediately pays the cost of cooperation, c. However, with probability q_1 the donor's reputation is updated, and cooperation gives him a good reputation with probability $(1-e_1)$ (=if an error does not occur). If the focal player does not leave the population (which occurs with probability ω), he can enjoy the advantage of a good reputation in future interactions, which is given by v. In sum, cooperation gives the donor the total benefit of $-c$ (immediate benefit) plus $q_1(1-e_1)\omega v$ (future benefit).

On the other hand, defection is costless (payoff $= 0$). However, with probability q_1 his reputation is updated, and defection gives him a good reputation only erroneously, with probability e_1. If the focal player does not leave the population (which occurs with probability ω), he is unlikely to enjoy the advantage of a good reputation, which marginal value is given by v. In sum, defection gives a

Table E: Calculation of the best action

action	evaluation	benefit: immediate	benefit: future	benefit: total	C is the better when...
C	G	$-c$	$q_i(1-e_i)\omega v$	$-c+q_i(1-e_i)\omega v$	$-c>0$
D	G	0	$q_i(1-e_i)\omega v$	$0+q_i(1-e_i)\omega v$	
C	G	$-c$	$q_i(1-e_i)\omega v$	$-c+q_i(1-e_i)\omega v$	$q_i(1-2e_i)\omega v>c$
D	B	0	$q_ie_i\omega v$	$0+q_ie_i\omega v$	
C	B	$-c$	$q_ie_i\omega v$	$-c+q_ie_i\omega v$	$-q_i(1-2e_i)\omega v>c$
D	G	0	$q_i(1-e_i)\omega v$	$0+q_i(1-e_i)\omega v$	
C	B	$-c$	$q_ie_i\omega v$	$-c+q_ie_i\omega v$	$-c>0$
D	B	0	$q_ie_i\omega v$	$0+q_ie_i\omega v$	

donor the total benefit of 0 (immediate benefit) plus $q_1e_1\omega v$ (future benefit).

The best action follows immediately by comparing the total benefit of cooperation (in this case, $-c+q_1(1-e_1)\omega v$) and that of defection ($=0+q_1e_1\omega v$). Whichever gives the larger value is the best action.

Note that in the argument above we have not considered future payoffs of the donor when the donor's reputation is not updated (which occurs with probability $1-q_1$). However, it suffices for our purpose as long as we are interested in the difference in payoff consequences between cooperation and defection, because the donor's current action yields no difference in future payoff consequences unless his reputation is updated.

These calculations are summarized in Table E. This procedure is repeated for each of the four columns in the social norm to derive the best action rule, a^*.

2.4 Reputation dynamics

To achieve our goal (ii) to estimate the equilibrium level cooperation performed in the population, here we derive the equilibrium frequency of good players. For that purpose, let us fix a pair of a social norm n and an action rule a and assume that all players adopt them. We denote by $g(t)$ the fraction of good players in the population at time t. In an infinitesimal time interval, Δt, a fraction $p_1\Delta t$ of players are chosen as a donor in public interactions and a fraction q_1 of them receive new reputations. Similarly, a fraction $p_2q_2\Delta t$ of players are chosen as a donor in private interactions and

receive new reputations. After a game interaction, a fraction ω of those who have acquired new reputations proceed to the next round, while the rest leaves the population and new players are supplied, whose initial reputations are determined by the average reputation in the population, as assumed before. The change in $g(t)$ is therefore described by the following differential equation:

$$\begin{aligned}\frac{\mathrm{d}g}{\mathrm{d}t} = & \underbrace{\omega p_1 q_1}_{\text{donors in public interactions}} \cdot \underbrace{\{gF_{1,\mathrm{G}} + (1-g)F_{1,\mathrm{B}}\}}_{\text{probability that their new reputation is good}} \\ + & \underbrace{\omega p_2 q_2}_{\text{donors in private interactions}} \cdot \underbrace{\{gF_{2,\mathrm{G}} + (1-g)F_{2,\mathrm{B}}\}}_{\text{probability that their new reputation is good}} \\ + & \underbrace{\omega(1-p_1q_1-p_2q_2)g}_{\text{good donors not subject to reputation update who proceed to the next round}} \\ + & \underbrace{(1-\omega)g}_{\text{newly supplied players}} \\ - & \underbrace{g}_{\text{good players who play donor}} ,\end{aligned} \tag{2.3}$$

where F's respectively represent the probability with which a player with action norm a receives a good reputation when they meet a good player in a public interaction ($F_{1,\mathrm{G}}$), when they meet a bad player in a public interaction ($F_{1,\mathrm{B}}$), when they meet a good player in a private interaction ($F_{2,\mathrm{G}}$), and when they meet a bad player in a private interaction ($F_{2,\mathrm{B}}$). Each of these F's is either $(1-e_i)$ $(i=1,2)$ (the action rule a prescribes a 'good' action under the social norm n, and the donor receives the correct reputation) or e_i $(i=1,2)$ (an action rule a prescribes a 'bad' action under the social norm n, but the donor erroneously receives a good reputation).

The equilibrium fraction of good players, g^*, is derived by setting the left-hand-side of eq.(2.3) as zero. It is not difficult to confirm that the resulting equation always has the unique solution, g^*.

Once we know g^*, it is straightforward to calculate the average level of cooperation at the equilibrium (=the fraction of interactions where the donor chooses cooperation out of all interactions), because the following four scenarios, meeting a good recipient in a public interaction, meeting a bad recipient in a public interaction, meeting a good recipient in a private interaction, and meeting a bad recipient in a private interaction, occur with probabilities $p_1g^*, p_1(1-g^*), p_2g^*$ and $p_2(1-g^*)$, respectively; the action rule a prescribes whether donors play cooperation or defection in each of the four scenarios.

3 Results of ESS search

3.1 Sign of marginal value

The analyses described in the previous section can be done for three separate cases, $v > 0$, $v = 0$ and $v < 0$. The easiest one is the case of $v = 0$, where a value of having a good reputation is null. Substituting $v = 0$ in Table E immediately tells us that no action rules other than action rule DDDD (=ALLD action rule) can be the best response. This shows that if $v = 0$ then action rules other than DDDD can never be evolutionarily stable strategies. Conversely, if the population is dominated by DDDD, from eq.(2.2) and Table D it immediately follows that $v = 0$ and that DDDD is evolutionarily stable under ANY social norms. This completes the analysis for the case of $v = 0$. However, because the fact that DDDD is evolutionarily stable is obvious, and because DDDD contributes nothing to cooperation, we will neglect the case of $v = 0$ in the following, unless otherwise specified.

To study the case of $v < 0$, we observe an interesting fact; that the labels 'good' and 'bad' are used only to distinguish two different social statuses of individuals in our model. Therefore, without loss of generality we can swap these two labels unless we are particularly interested in their meaning. Therefore, whenever we find that v is negative, we can make it positive without changing its absolute value by swapping 'good' and 'bad' (it is called mirror symmetry: see [12]). Rather, we believe that it should be natural to do so, because 'good' status usually suggests some sort of advantage over 'bad' status. Hence, in the following we present the results of our search only for $v > 0$ only.

3.2 Notations

We use the table-format shown in Table F to show the combinations of a social norm and an action rule we found in our exhaustive search, where the action rule described is ESS under the social norm described. Tables read as follows:

ESS Code labels the combination of a social norm and an action rule we found, in the format of (arabic number)-(Greek alphabet), where (arabic number) encodes distinct social norms and (Greek alphabet) encodes distinct action rules.

social norm describes the social norm that makes non-ALLD action rules evolutionarily stable. Asterisks (*) represent wild-cards; they can be either G or B.

action rule describes the action rule that is ESS under the social norm.

invader describes the mutant action rule that potentially invades the ESS action rule when the ESS condition is violated.

ESS condition describes the condition for the action norm to be evolutionarily stable. If the condition consists of more than one inequalities, corresponding potential invaders are shown in the 'invader' cell in the same order as these inequalities (except inequalities inside parentheses).

G-level describes the equilibrium level of good players in the population ($= g^*$)

C-level describes the equilibrium level of cooperation in the population

We will use the following notations for error rates: $\hat{e}_1 = 1 - 2e_1, \hat{e}_2 = 1 - 2e_2$.

Table F: A format for displaying ESS

ESS Code							
situation & partner		public good	public bad	private good	private bad	ESS	ESS condition
social norm	C	**G/B**	**G/B**	**G/B**	**G/B**	G-level	eq'm fraction of good players ($= g^*$)
	D	**G/B**	**G/B**	**G/B**	**G/B**		
action rule		**C/D**	**C/D**	**C/D**	**C/D**	C-level	eq'm fraction of cooperative interactions
invader		potential invaders					

3.3 Full list

ESS 1α							
situation & partner		public good	public bad	private good	private bad	ESS	$q_2\hat{e}_2\omega > \frac{c}{b}\frac{2-\omega(2-\bar{q})}{p_2}$
social norm	C	**G**	**G**	**G**	*	G-level	$\frac{(1-e_1)p_1q_1 + (1-e_2)p_2q_2}{p_1q_1 + p_2q_2}$
	D	**B**	**B**	**B**	**G**		
action rule		**C**	**C**	**C**	**D**	C-level	$p_1 + g^*p_2$
invader		CCDD					

ESS 2α							
situation & partner		public		private		ESS	$q_2\hat{e}_2\omega > \frac{c}{b}\frac{2-\omega(2-\bar{q})}{p_2}$
		good	bad	good	bad		
social norm	C	**G**	**G**	**G**	**B**	G-level	$\frac{(1-e_1)p_1q_1+e_2p_2q_2}{p_1q_1+2e_2p_2q_2}$
	D	**B**	**B**	**B**	**B**		
action rule		**C**	**C**	**C**	**D**	C-level	$p_1+g^*p_2$
invader		CCDD					

ESS 3β							
situation & partner		public		private		ESS	$q_2\hat{e}_2\omega > \frac{c}{b}\frac{2-\omega(2-\bar{q})}{p_1}$
		good	bad	good	bad		
social norm	C	**G**	*	**G**	**G**	G-level	$\frac{(1-e_1)p_1q_1+(1-e_2)p_2q_2}{p_1q_1+p_2q_2}$
	D	**B**	**G**	**B**	**B**		
action rule		**C**	**D**	**C**	**C**	C-level	$g^*p_1+p_2$
invader		CDDD					

ESS 3ϵ							
situation & partner		public		private		ESS	$q_1\hat{e}_1\omega > \frac{c}{b}\frac{2-\omega(2-\bar{q})}{p_1} > q_2\hat{e}_2\omega$
		good	bad	good	bad		
social norm	C	**G**	*	**G**	**G**	G-level	$\frac{(1-e_1)p_1q_1+e_2p_2q_2}{p_1q_1+p_2q_2}$
	D	**B**	**G**	**B**	**B**		
action rule		**C**	**D**	**D**	**D**	C-level	g^*p_1
invader		DDDD,CDCC					

ESS 4β							
situation & partner		public		private		ESS	$q_2\hat{e}_2\omega > \frac{c}{b}\frac{2-\omega(2-\bar{q})}{p_1}$
		good	bad	good	bad		
social norm	C	**G**	**B**	**G**	**G**	G-level	$\frac{e_1p_1q_1+(1-e_2)p_2q_2}{2e_1p_1q_1+p_2q_2}$
	D	**B**	**B**	**B**	**B**		
action rule		**C**	**D**	**C**	**C**	C-level	$g^*p_1+p_2$
invader		CDDD					

ESS 4ϵ							
situation & partner		public		private		ESS	$q_1\hat{e}_1\omega > \frac{c}{b}\frac{2-\omega(2-\bar{q})}{p_1} > q_2\hat{e}_2\omega$
		good	bad	good	bad		
social norm	C	**G**	**B**	**G**	**G**	G-level	$\frac{e_1p_1q_1+e_2p_2q_2}{2e_1p_1q_1+p_2q_2}$
	D	**B**	**B**	**B**	**B**		
action rule		**C**	**D**	**D**	**D**	C-level	g^*p_1
invader		DDDD,CDCC					

ESS 5γ							
situation & partner		public		private		ESS	$q_2\hat{e}_2\omega > \frac{c}{b}[2-\omega(2-\bar{q})]$
		good	bad	good	bad		
social norm	C	**G**	*	**G**	*	G-level	$\frac{(1-e_1)p_1q_1+(1-e_2)p_2q_2}{p_1q_1+p_2q_2}$
	D	**B**	**G**	**B**	**G**		
action rule		**C**	**D**	**C**	**D**	C-level	g^*
invader		CDDD					

ESS 5ϵ							
situation & partner		public		private		ESS	$q_1\hat{e}_1\omega > \frac{c}{b}\frac{2-\omega(2-\bar{q})}{p_1} > q_2\hat{e}_2\omega$
		good	bad	good	bad		
social norm	C	**G**	*	**G**	*	G-level	$\frac{(1-e_1)p_1q_1+(1-e_2)p_2q_2}{p_1q_1+2(1-e_2)p_2q_2}$
	D	**B**	**G**	**B**	**G**		
action rule		**C**	**D**	**D**	**D**	C-level	g^*p_1
invader		DDDD,CDCD					

ESS 6γ							
situation & partner		public		private		ESS	$q_2\hat{e}_2\omega > \frac{c}{b}[2-\omega(2-\bar{q})]$
		good	bad	good	bad		
social norm	C	**G**	*	**G**	**B**	G-level	$\frac{(1-e_1)p_1q_1+e_2p_2q_2}{p_1q_1+2e_2p_2q_2}$
	D	**B**	**G**	**B**	**B**		
action rule		**C**	**D**	**C**	**D**	C-level	g^*
invader		CDDD					

ESS 6ϵ							
situation & partner		public		private		ESS	$q_1\hat{e}_1\omega > \frac{c}{b}\frac{2-\omega(2-\bar{q})}{p_1} > q_2\hat{e}_2\omega$
		good	bad	good	bad		
social norm	C	**G**	*	**G**	**B**	G-level	$\frac{(1-e_1)p_1q_1+e_2p_2q_2}{p_1q_1+p_2q_2}$
	D	**B**	**G**	**B**	**B**		
action rule		**C**	**D**	**D**	**D**	C-level	g^*p_1
invader		DDDD,CDCD					

ESS 7γ							
situation & partner		public		private		ESS	$q_2\hat{e}_2\omega > \frac{c}{b}[2-\omega(2-\bar{q})]$
		good	bad	good	bad		
social norm	C	**G**	**B**	**G**	*	G-level	$\frac{e_1p_1q_1+(1-e_2)p_2q_2}{2e_1p_1q_1+p_2q_2}$
	D	**B**	**B**	**B**	**G**		
action rule		**C**	**D**	**C**	**D**	C-level	g^*
invader		CDDD					

ESS 7ϵ							
situation & partner		public		private		ESS	$q_1\hat{e}_1\omega > \frac{c}{b}\frac{2-\omega(2-\bar{q})}{p_1} > q_2\hat{e}_2\omega$
		good	bad	good	bad		
social norm	C	**G**	**B**	**G**	*	G-level	$\frac{1}{2}$
	D	**B**	**B**	**B**	**G**		
action rule		**C**	**D**	**D**	**D**	C-level	$g^* p_1 = \frac{1}{2}p_1$
invader		DDDD,CDCD					

ESS 8γ							
situation & partner		public		private		ESS	$q_2\hat{e}_2\omega > \frac{c}{b}[2-\omega(2-\bar{q})]$
		good	bad	good	bad		
social norm	C	**G**	**B**	**G**	**B**	G-level	$\frac{1}{2}$
	D	**B**	**B**	**B**	**B**		
action rule		**C**	**D**	**C**	**D**	C-level	$g^* = \frac{1}{2}$
invader		CDDD					

ESS 8ϵ							
situation & partner		public		private		ESS	$q_1\hat{e}_1\omega > \frac{c}{b}\frac{2-\omega(2-\bar{q})}{p_1} > q_2\hat{e}_2\omega$
		good	bad	good	bad		
social norm	C	**G**	**B**	**G**	**B**	G-level	$\frac{e_1p_1q_1 + e_2p_2q_2}{2e_1p_1q_1 + p_2q_2}$
	D	**B**	**B**	**B**	**B**		
action rule		**C**	**D**	**D**	**D**	C-level	$g^* p_1$
invader		DDDD,CDCD					

ESS 9δ							
situation & partner		public		private		ESS	$q_2\hat{e}_2\omega > \frac{c}{b}\frac{2-\omega(2-\bar{q})}{p_1-p_2}$ $(p_1 > p_2)$
		good	bad	good	bad		
social norm	C	**G**	*	*	**G**	G-level	$\frac{(1-e_1)p_1q_1 + (1-e_2)p_2q_2}{p_1q_1 + p_2q_2}$
	D	**B**	**G**	**G**	**B**		
action rule		**C**	**D**	**D**	**C**	C-level	$g^* p_1 + (1-g^*)p_2$
invader		CDDD					

ESS 9ϵ							
situation & partner		public		private		ESS	$q_1\hat{e}_1\omega > \frac{c}{b}\frac{2-\omega(2-\bar{q})}{p_1} > q_2\hat{e}_2\omega$
		good	bad	good	bad		
social norm	C	**G**	*	*	**G**	G-level	$\frac{(1-e_1)p_1q_1 + e_2p_2q_2}{p_1q_1 + 2e_2p_2q_2}$
	D	**B**	**G**	**G**	**B**		
action rule		**C**	**D**	**D**	**D**	C-level	$g^* p_1$
invader		DDDD,CDDC					

ESS 10δ							
situation & partner		public		private		ESS	$q_2\hat{e}_2\omega > \frac{c}{b}\frac{2-\omega(2-\bar{q})}{p_1-p_2} \quad (p_1 > p_2)$
		good	bad	good	bad		
social norm	C	**G**	*	**B**	**G**	G-level	$\frac{(1-e_1)p_1q_1+(1-e_2)p_2q_2}{p_1q_1+2(1-e_2)p_2q_2}$
	D	**B**	**G**	**B**	**B**		
action rule		**C**	**D**	**D**	**C**	C-level	$g^*p_1+(1-g^*)p_2$
invader		CDDD					

ESS 10ϵ							
situation & partner		public		private		ESS	$q_1\hat{e}_1\omega > \frac{c}{b}\frac{2-\omega(2-\bar{q})}{p_1} > q_2\hat{e}_2\omega$
		good	bad	good	bad		
social norm	C	**G**	*	**B**	**G**	G-level	$\frac{(1-e_1)p_1q_1+e_2p_2q_2}{p_1q_1+p_2q_2}$
	D	**B**	**G**	**B**	**B**		
action rule		**C**	**D**	**D**	**D**	C-level	g^*p_1
invader		DDDD,CDDC					

ESS 11δ							
situation & partner		public		private		ESS	$q_2\hat{e}_2\omega > \frac{c}{b}\frac{2-\omega(2-\bar{q})}{p_1-p_2} \quad (p_1 > p_2)$
		good	bad	good	bad		
social norm	C	**G**	**B**	*	**G**	G-level	$\frac{e_1p_1q_1+(1-e_2)p_2q_2}{2e_1p_1q_1+p_2q_2}$
	D	**B**	**B**	**G**	**B**		
action rule		**C**	**D**	**D**	**C**	C-level	$g^*p_1+(1-g^*)p_2$
invader		CDDD					

ESS 11ϵ							
situation & partner		public		private		ESS	$q_1\hat{e}_1\omega > \frac{c}{b}\frac{2-\omega(2-\bar{q})}{p_1} > q_2\hat{e}_2\omega$
		good	bad	good	bad		
social norm	C	**G**	**B**	*	**G**	G-level	$\frac{1}{2}$
	D	**B**	**B**	**G**	**B**		
action rule		**C**	**D**	**D**	**D**	C-level	$g^*p_1 = \frac{1}{2}p_1$
invader		DDDD,CDDC					

ESS 12δ							
situation & partner		public		private		ESS	$q_2\hat{e}_2\omega > \frac{c}{b}\frac{2-\omega(2-\bar{q})}{p_1-p_2} \quad (p_1 > p_2)$
		good	bad	good	bad		
social norm	C	**G**	**B**	**B**	**G**	G-level	$\frac{1}{2}$
	D	**B**	**B**	**B**	**B**		
action rule		**C**	**D**	**D**	**C**	C-level	$g^*p_1+(1-g^*)p_2 = \frac{1}{2}$
invader		CDDD					

ESS 12ϵ							
situation & partner		public		private		ESS	$q_1\hat{e}_1\omega > \frac{c}{b}\frac{2-\omega(2-\bar{q})}{p_1} > q_2\hat{e}_2\omega$
		good	bad	good	bad		
social norm	C	**G**	**B**	**B**	**G**	G-level	$\frac{e_1p_1q_1+e_2p_2q_2}{2e_1p_1q_1+p_2q_2}$
	D	**B**	**B**	**B**	**B**		
action rule		**C**	**D**	**D**	**D**	C-level	g^*p_1
invader		DDDD,CDDC					

ESS 13ϵ							
situation & partner		public		private		ESS	$q_1\hat{e}_1\omega > \frac{c}{b}\frac{2-\omega(2-\bar{q})}{p_1}$
		good	bad	good	bad		
social norm	C	**G**	*	*	*	G-level	$\frac{(1-e_1)p_1q_1+(1-e_2)p_2q_2}{p_1q_1+p_2q_2}$
	D	**B**	**G**	**G**	**G**		
action rule		**C**	**D**	**D**	**D**	C-level	g^*p_1
invader		DDDD					

ESS 14ϵ							
situation & partner		public		private		ESS	$q_1\hat{e}_1\omega > \frac{c}{b}\frac{2-\omega(2-\bar{q})}{p_1}$
		good	bad	good	bad		
social norm	C	**G**	*	*	**B**	G-level	$\frac{(1-e_1)p_1q_1+e_2p_2q_2}{p_1q_1+2e_2p_2q_2}$
	D	**B**	**G**	**G**	**B**		
action rule		**C**	**D**	**D**	**D**	C-level	g^*p_1
invader		DDDD					

ESS 15ϵ							
situation & partner		public		private		ESS	$q_1\hat{e}_1\omega > \frac{c}{b}\frac{2-\omega(2-\bar{q})}{p_1}$
		good	bad	good	bad		
social norm	C	**G**	*	**B**	*	G-level	$\frac{(1-e_1)p_1q_1+(1-e_2)p_2q_2}{p_1q_1+2(1-e_2)p_2q_2}$
	D	**B**	**G**	**B**	**G**		
action rule		**C**	**D**	**D**	**D**	C-level	g^*p_1
invader		DDDD					

ESS 16ϵ							
situation & partner		public		private		ESS	$q_1\hat{e}_1\omega > \frac{c}{b}\frac{2-\omega(2-\bar{q})}{p_1}$
		good	bad	good	bad		
social norm	C	**G**	*	**B**	**B**	G-level	$\frac{(1-e_1)p_1q_1+e_2p_2q_2}{p_1q_1+p_2q_2}$
	D	**B**	**G**	**B**	**B**		
action rule		**C**	**D**	**D**	**D**	C-level	g^*p_1
invader		DDDD					

ESS 17ϵ							
situation & partner		public		private		ESS	$q_1\hat{e}_1\omega > \frac{c}{b}\frac{2-\omega(2-\bar{q})}{p_1}$
		good	bad	good	bad		
social norm	C	**G**	**B**	*	*	G-level	$\frac{e_1p_1q_1+(1-e_2)p_2q_2}{2e_1p_1q_1+p_2q_2}$
	D	**B**	**B**	**G**	**G**		
action rule		**C**	**D**	**D**	**D**	C-level	g^*p_1
invader		DDDD					

ESS 18ϵ							
situation & partner		public		private		ESS	$q_1\hat{e}_1\omega > \frac{c}{b}\frac{2-\omega(2-\bar{q})}{p_1}$
		good	bad	good	bad		
social norm	C	**G**	**B**	*	**B**	G-level	$\frac{1}{2}$
	D	**B**	**B**	**G**	**B**		
action rule		**C**	**D**	**D**	**D**	C-level	$g^*p_1 = \frac{1}{2}p_1$
invader		DDDD					

ESS 19ϵ							
situation & partner		public		private		ESS	$q_1\hat{e}_1\omega > \frac{c}{b}\frac{2-\omega(2-\bar{q})}{p_1}$
		good	bad	good	bad		
social norm	C	**G**	**B**	**B**	*	G-level	$\frac{1}{2}$
	D	**B**	**B**	**B**	**G**		
action rule		**C**	**D**	**D**	**D**	C-level	$g^*p_1 = \frac{1}{2}p_1$
invader		DDDD					

ESS 20ϵ							
situation & partner		public		private		ESS	$q_1\hat{e}_1\omega > \frac{c}{b}\frac{2-\omega(2-\bar{q})}{p_1}$
		good	bad	good	bad		
social norm	C	**G**	**B**	**B**	**B**	G-level	$\frac{e_1p_1q_1+e_2p_2q_2}{2e_1p_1q_1+p_2q_2}$
	D	**B**	**B**	**B**	**B**		
action rule		**C**	**D**	**D**	**D**	C-level	g^*p_1
invader		DDDD					

ESS 21ζ							
situation & partner		public		private		ESS	$q_2\hat{e}_2\omega > \frac{c}{b}\frac{2-\omega(2-\bar{q})}{p_2-p_1} \quad (p_1 < p_2)$
		good	bad	good	bad		
social norm	C	*	**G**	**G**	*	G-level	$\frac{(1-e_1)p_1q_1+(1-e_2)p_2q_2}{p_1q_1+p_2q_2}$
	D	**G**	**B**	**B**	**G**		
action rule		**D**	**C**	**C**	**D**	C-level	$(1-g^*)p_1+g^*p_2$
invader		DCDD					

ESS 22ζ							
situation & partner		public		private		ESS	$q_2\hat{e}_2\omega > \frac{c}{b}\frac{2-\omega(2-\bar{q})}{p_2-p_1}$ $(p_1 < p_2)$
		good	bad	good	bad		
social norm	C	*	**G**	**G**	**B**	G-level	$\frac{(1-e_1)p_1q_1+e_2p_2q_2}{p_1q_1+2e_2p_2q_2}$
	D	**G**	**B**	**B**	**B**		
action rule		**D**	**C**	**C**	**D**	C-level	$(1-g^*)p_1+g^*p_2$
invader		DCDD					

ESS 23ζ							
situation & partner		public		private		ESS	$q_2\hat{e}_2\omega > \frac{c}{b}\frac{2-\omega(2-\bar{q})}{p_2-p_1}$ $(p_1 < p_2)$
		good	bad	good	bad		
social norm	C	**B**	**G**	**G**	*	G-level	$\frac{(1-e_1)p_1q_1+(1-e_2)p_2q_2}{2(1-e_1)p_1q_1+p_2q_2}$
	D	**B**	**B**	**B**	**G**		
action rule		**D**	**C**	**C**	**D**	C-level	$(1-g^*)p_1+g^*p_2$
invader		DCDD					

ESS 24ζ							
situation & partner		public		private		ESS	$q_2\hat{e}_2\omega > \frac{c}{b}\frac{2-\omega(2-\bar{q})}{p_2-p_1}$ $(p_1 < p_2)$
		good	bad	good	bad		
social norm	C	**B**	**G**	**G**	**B**	G-level	$\frac{1}{2}$
	D	**B**	**B**	**B**	**B**		
action rule		**D**	**C**	**C**	**D**	C-level	$(1-g^*)p_1+g^*p_2=\frac{1}{2}$
invader		DCDD					

ESS 25η							
situation & partner		public		private		ESS	$q_2\hat{e}_2\omega > \frac{c}{b}\frac{2-\omega(2-\bar{q})}{p_2}$
		good	bad	good	bad		
social norm	C	*	*	**G**	*	G-level	$\frac{(1-e_1)p_1q_1+(1-e_2)p_2q_2}{p_1q_1+p_2q_2}$
	D	**G**	**G**	**B**	**G**		
action rule		**D**	**D**	**C**	**D**	C-level	g^*p_2
invader		DDDD					

ESS 26η							
situation & partner		public		private		ESS	$q_2\hat{e}_2\omega > \frac{c}{b}\frac{2-\omega(2-\bar{q})}{p_2}$
		good	bad	good	bad		
social norm	C	*	*	**G**	**B**	G-level	$\frac{(1-e_1)p_1q_1+e_2p_2q_2}{p_1q_1+2e_2p_2q_2}$
	D	**G**	**G**	**B**	**B**		
action rule		**D**	**D**	**C**	**D**	C-level	g^*p_2
invader		DDDD					

ESS 27η							
situation & partner		public		private		ESS	$q_2\hat{e}_2\omega > \frac{c}{b}\frac{2-\omega(2-\bar{q})}{p_2}$
		good	bad	good	bad		
social norm	C	*	**B**	**G**	*	G-level	$\frac{e_1p_1q_1+(1-e_2)p_2q_2}{2e_1p_1q_1+p_2q_2}$
	D	**G**	**B**	**B**	**G**		
action rule		**D**	**D**	**C**	**D**	C-level	g^*p_2
invader		DDDD					

ESS 28η							
situation & partner		public		private		ESS	$q_2\hat{e}_2\omega > \frac{c}{b}\frac{2-\omega(2-\bar{q})}{p_2}$
		good	bad	good	bad		
social norm	C	*	**B**	**G**	**B**	G-level	$\frac{1}{2}$
	D	**G**	**B**	**B**	**B**		
action rule		**D**	**D**	**C**	**D**	C-level	$g^*p_2 = \frac{1}{2}p_2$
invader		DDDD					

ESS 29η							
situation & partner		public		private		ESS	$q_2\hat{e}_2\omega > \frac{c}{b}\frac{2-\omega(2-\bar{q})}{p_2}$
		good	bad	good	bad		
social norm	C	**B**	*	**G**	*	G-level	$\frac{(1-e_1)p_1q_1+(1-e_2)p_2q_2}{2(1-e_1)p_1q_1+p_2q_2}$
	D	**B**	**G**	**B**	**G**		
action rule		**D**	**D**	**C**	**D**	C-level	g^*p_2
invader		DDDD					

ESS 30η							
situation & partner		public		private		ESS	$q_2\hat{e}_2\omega > \frac{c}{b}\frac{2-\omega(2-\bar{q})}{p_2}$
		good	bad	good	bad		
social norm	C	**B**	*	**G**	**B**	G-level	$\frac{1}{2}$
	D	**B**	**G**	**B**	**B**		
action rule		**D**	**D**	**C**	**D**	C-level	$g^*p_2 = \frac{1}{2}p_2$
invader		DDDD					

ESS 31η							
situation & partner		public		private		ESS	$q_2\hat{e}_2\omega > \frac{c}{b}\frac{2-\omega(2-\bar{q})}{p_2}$
		good	bad	good	bad		
social norm	C	**B**	**B**	**G**	*	G-level	$\frac{e_1p_1q_1+(1-e_2)p_2q_2}{p_1q_1+p_2q_2}$
	D	**B**	**B**	**B**	**G**		
action rule		**D**	**D**	**C**	**D**	C-level	g^*p_2
invader		DDDD					

ESS 32η							
situation & partner		public		private		ESS	$q_2\hat{e}_2\omega > \frac{c}{b}\frac{2-\omega(2-\bar{q})}{p_2}$
		good	bad	good	bad		
social norm	C	**B**	**B**	**G**	**B**	G-level	$\frac{e_1p_1q_1+e_2p_2q_2}{p_1q_1+2e_2p_2q_2}$
	D	**B**	**B**	**B**	**B**		
action rule		**D**	**D**	**C**	**D**	C-level	g^*p_2
invader		DDDD					

3.4 Classification of ESS

As described above, we found 32 (87 without using wild-cards) different types of social norms that can make action rules than DDDD evolutionarily stable. 10 (21 without wild-cards) of them make multiple (=two) action rules other than DDDD evolutionarily stable (norms 3 to 12). There are 7 action rules (each labeled as $\alpha, \beta, \gamma, \delta, \epsilon, \zeta$ and η) that are not DDDD and that become ESS under specific social norms, and they are summarized below. Note that dagger marks (†) below are special wild-cards; the corresponding column can be either GG, BG, or BB (only GB is not allowed).

α =CCCD; $1\alpha, 2\alpha$							
situation & partner		public		private		ESS	$q_2\hat{e}_2\omega > \frac{c}{b}\frac{2-\omega(2-\bar{q})}{p_2}$
		good	bad	good	bad		
social norm	C	**G**	**G**	**G**	†		
	D	**B**	**B**	**B**	†		
action rule		**C**	**C**	**C**	**D**		
invader		CCDD					

β =CDCC; $3\beta, 4\beta$							
situation & partner		public		private		ESS	$q_2\hat{e}_2\omega > \frac{c}{b}\frac{2-\omega(2-\bar{q})}{p_1}$
		good	bad	good	bad		
social norm	C	**G**	†	**G**	**G**		
	D	**B**	†	**B**	**B**		
action rule		**C**	**D**	**C**	**C**		
invader		CDDD					

γ =CDCD (honest); $5\gamma, 6\gamma, 7\gamma, 8\gamma$							
situation & partner		public		private		ESS	$q_2\hat{e}_2\omega > \frac{c}{b}[2-\omega(2-\bar{q})]$
		good	bad	good	bad		
social norm	C	**G**	†	**G**	†		
	D	**B**	†	**B**	†		
action rule		**C**	**D**	**C**	**D**		
invader		CDDD					

δ =CDDC; $9\delta, 10\delta, 11\delta, 12\delta$							
situation & partner		public		private		ESS	$q_2\hat{e}_2\omega > \frac{c}{b}\frac{2-\omega(2-\bar{q})}{p_1-p_2} \quad (p_1 > p_2)$
		good	bad	good	bad		
social norm	C	**G**	†	†	**G**		
	D	**B**	†	†	**B**		
action rule		**C**	**D**	**D**	**C**		
invader		CDDD					

ϵ =CDDD (hypocrite); $3\epsilon, 4\epsilon$							
situation & partner		public		private		ESS	$q_1\hat{e}_1\omega > \frac{c}{b}\frac{2-\omega(2-\bar{q})}{p_1} > q_2\hat{e}_2\omega$
		good	bad	good	bad		
social norm	C	**G**	†	**G**	**G**		
	D	**B**	†	**B**	**B**		
action rule		**C**	**D**	**D**	**D**		
invader		DDDD, CDCC					

ϵ =CDDD (hypocrite); $5\epsilon, 6\epsilon, 7\epsilon, 8\epsilon$							
situation & partner		public		private		ESS	$q_1\hat{e}_1\omega > \frac{c}{b}\frac{2-\omega(2-\bar{q})}{p_1} > q_2\hat{e}_2\omega$
		good	bad	good	bad		
social norm	C	**G**	†	**G**	†		
	D	**B**	†	**B**	†		
action rule		**C**	**D**	**D**	**D**		
invader		DDDD, CDCD					

ϵ =CDDD (hypocrite); $9\epsilon, 10\epsilon, 11\epsilon, 12\epsilon$							
situation & partner		public		private		ESS	$q_1\hat{e}_1\omega > \frac{c}{b}\frac{2-\omega(2-\bar{q})}{p_1} > q_2\hat{e}_2\omega$
		good	bad	good	bad		
social norm	C	**G**	†	†	**G**		
	D	**B**	†	†	**B**		
action rule		**C**	**D**	**D**	**D**		
invader		DDDD, CDDC					

ϵ =CDDD (hypocrite); $13\epsilon, 14\epsilon, 15\epsilon, 16\epsilon, 17\epsilon, 18\epsilon, 19\epsilon, 20\epsilon$							
situation & partner		public		private		ESS	$q_1\hat{e}_1\omega > \frac{c}{b}\frac{2-\omega(2-\bar{q})}{p_1}$
		good	bad	good	bad		
social norm	C	**G**	†	†	†		
	D	**B**	†	†	†		
action rule		**C**	**D**	**D**	**D**		
invader		DDDD					

ζ =DCCD; $21\zeta, 22\zeta, 23\zeta, 24\zeta$							
situation & partner		public		private		ESS	$q_2\hat{e}_2\omega > \frac{c}{b}\frac{2-\omega(2-\bar{q})}{p_2-p_1} \quad (p_1 < p_2)$
		good	bad	good	bad		
social norm	C	†	**G**	**G**	†		
	D	†	**B**	**B**	†		
action rule		**D**	**C**	**C**	**D**		
invader		DCDD					

η =DDCD; $25\eta, 26\eta, 27\eta, 28\eta, 29\eta, 30\eta, 31\eta, 32\eta$							
situation & partner		public		private		ESS	$q_2\hat{e}_2\omega > \frac{c}{b}\frac{2-\omega(2-\bar{q})}{p_2}$
		good	bad	good	bad		
social norm	C	†	†	**G**	†		
	D	†	†	**B**	†		
action rule		**D**	**D**	**C**	**D**		
invader		DDDD					

3.5 Equilibrium fraction of good players

In the small-error limit, $e_1, e_2 \to 0$, the equilibrium fraction of good players, g^*, converges to unity for the following 18 types of ESS: $1\alpha, 2\alpha, 3\beta, 4\beta, 5\gamma, 6\gamma, 7\gamma, 9\delta, 9\epsilon, 11\delta, 13\epsilon, 14\epsilon, 17\epsilon, 21\zeta, 22\zeta, 25\eta, 26\eta$ and 27η.

3.6 Equilibrium level of cooperation

We are particularly interested in the equilibrium level of cooperation in indirect reciprocity interactions. For that purpose, consider the small-error limit $e_1, e_2 \to 0$. We find that in this limit the following seven types of ESS achieve full cooperation: $1\alpha, 2\alpha, 3\beta, 4\beta, 5\gamma, 6\gamma$ and 7γ.

4 Reduced Model in the Main Text

4.1 Model parameters

So far we have studied a full model of indirect reciprocity with $m = 2$ situations, where our parameters are b, c (benefit and cost of cooperation) p_1, p_2 (probability of occurrence of public and private situations, $p_1 + p_2 = 1$), q_1, q_2 (observability there), e_1, e_2 (error rates there), and ω (continuation probability).

Herein we study a reduce model, where we assume perfect observability in public interactions ($q_1 = 1$ and $q_2 = q$), negligibly small (but positive) error rates ($e_1, e_2 \to 0$), and a large number of

game interactions per player ($\omega \to 1$). We also simply refer to p_1 as p (and therefore $p_2 = 1 - p$). Thus we obtain a four-parameter model with b, c (benefit and cost of cooperation), p (probability of occurrence of a public situation), and q (observability in private situations). This simplification is summarized in Table G. In the following we study $0 < p, q < 1$.

Table G: Parameters in the reduced model

full model	reduced model
b	b
c	c
p_1	p
p_2	$1 - p$
q_1	1
q_2	q
e_1	$\to 0$
e_2	$\to 0$
ω	$\to 1$

4.2 ESS search

Based on the ESS search and ESS classification for the full model in Section 3, we herein study the reduced model above to find ESS which achieves the highest level of cooperation among all ESS.

For a given set of parameters, b, c, p and q, the ESS condition for each type of ESS is given as

$$\begin{cases} \dfrac{b}{c} > \dfrac{\bar{q}}{(1-p)q} & \text{(for action rule } \alpha = \text{CCCD)} \\ \dfrac{b}{c} > \dfrac{\bar{q}}{pq} & \text{(for action rule } \beta = \text{CDCC)} \\ \dfrac{b}{c} > \dfrac{\bar{q}}{q} & \text{(for action rule } \gamma = \text{CDCD)} \\ \dfrac{b}{c} > \dfrac{\bar{q}}{(2p-1)q} \quad \left(p > \dfrac{1}{2}\right) & \text{(for action rule } \delta = \text{CDDC)} \\ \dfrac{\bar{q}}{pq} > \dfrac{b}{c} > \dfrac{\bar{q}}{p} & \text{(for action rule } \epsilon = \text{CDDD; social norms } 3\epsilon \text{ to } 12\epsilon) \\ \dfrac{b}{c} > \dfrac{\bar{q}}{p} & \text{(for action rule } \epsilon = \text{CDDD; social norms } 13\epsilon \text{ to } 20\epsilon) \\ \dfrac{b}{c} > \dfrac{\bar{q}}{(1-2p)q} \quad \left(p < \dfrac{1}{2}\right) & \text{(for action rule } \zeta = \text{DCCD)} \\ \dfrac{b}{c} > \dfrac{\bar{q}}{(1-p)q} & \text{(for action rule } \eta = \text{DDCD),} \end{cases} \tag{4.4}$$

where $\bar{q} = p + (1-p)q$.

According to the classification in Section 3.6, ESS $1\alpha, 2\alpha, 3\beta, 4\beta, 5\gamma, 6\gamma$ and 7γ achieve full cooperation as long as they satisfy their ESS conditions. Therefore, if $b/c > \bar{q}/[(1-p)q]$ holds ESS 1α and 2α are some of the most cooperative ESS, if $b/c > \bar{q}/pq$ holds ESS 3β and 4β are some of the most cooperative ESS, and if $b/c > \bar{q}/q$ holds ESS $5\gamma, 6\gamma$ and 7γ are some of the most cooperative ESS. Because the ESS conditions for action rules δ =CDDC, ζ =DCCD, and η =DDCD are more strict that that of γ =CDCD, and because none of δ, ζ, η achieves full cooperation, they can never be the most cooperative ESS.

In contrast, if $b/c < \bar{q}/q$ holds, action rule ϵ =CDDD is the only ESS as long as $b/c > \bar{q}/p$. In that case, the equilibrium level of cooperation is not unity, but is equal to g^*p, where g^* represents the equilibrium fraction of good players (see Section 3.3). According to the analysis in Section 3.5, it becomes maximal ($g^* = 1$, and therefore $g^*p = p$) for ESS $9\epsilon, 13\epsilon$, and 14ϵ. Therefore we conclude that if $\bar{q}/q > b/c > \bar{q}/p$ holds ESS $9\epsilon, 13\epsilon$, and 14ϵ are the most cooperative ESS, and the equilibrium cooperation level equals to p.

Finally, if $b/c < \min\{\bar{q}/q, \bar{q}/p\}$ holds, action rule DDDD is the only possible ESS; no other ESS are found. Obviously the equilibrium cooperation level there is zero.

The result of our analysis of the reduced model is summarized in Table H.

Table H: Analytical results for the reduced model

condition	ESS type	eq'm level of cooperation
$\frac{b}{c} > \frac{\bar{q}}{q}$	CDCD$(5\gamma, 6\gamma, 7\gamma)$	1
	$\left(+\text{CCCD}(1\alpha, 2\alpha) \text{ if } \frac{b}{c} > \frac{\bar{q}}{(1-p)q} \right)$	
	$\left(+\text{CDCC}(3\beta, 4\beta) \text{ if } \frac{b}{c} > \frac{\bar{q}}{pq} \right)$	
$\frac{\bar{q}}{q} > \frac{b}{c} > \frac{\bar{q}}{p}$	CDDD$(9\epsilon, 13\epsilon, 14\epsilon)$	p
$\min\left\{\frac{\bar{q}}{q}, \frac{\bar{q}}{p}\right\} > \frac{b}{c}$	DDDD + any norm	0

4.3 Intuitions behind some ESS conditions

ESS conditions given in eq.(4.4) have intuitive explanations.

Consider the action rule α =CCCD. The most dangerous mutant is CCDD. To understand the ESS condition, $b/c > \bar{q}[(1-p)q]$, imagine a donor with a good reputation meeting a good recipient in a private interaction. Under social norms that make CCCD evolutionarily stable, if such a donor cooperates he pays the immediate cost of c but retains his good reputation. If the donor, on the other hand, defects, he pays no immediate cost but may lose his good reputation with probability q. In order for him to recover his good reputation, his must cooperate in a subsequent round (because all players are good under ESS 1α and 2α; see Section 3.5) and must be observed by a third party (that occurs with probability $\bar{q}$), so he will stay bad for $1/\bar{q}$ rounds of game interactions on average. The disadvantage of having a bad reputation compared with a good one is that a bad reputation holder misses receiving cooperation in a private situation from a CCCD donor, which imposes the loss of $(1-p)b$ per round on him. Therefore the expected total cost of defection is $q(1-p)b/\bar{q}$. In sum, cooperation pays when meeting a good recipient in a private interaction if $c < q(1-p)b/\bar{q}$ holds, which reproduces the ESS condition.

A similar reasoning applies to the action rule β =CDCC and its ESS condition, $b/c > \bar{q}/pq$. The

most dangerous mutant is CDDD. Imagine a donor with a good reputation meeting a recipient in a private interaction. Cooperation costs c but keeps the donor's good reputation, while defection costs none but may give him a bad reputation to with probability q. It takes $1/\bar{q}$ rounds on average for the donor to recover a good reputation. The disadvantage of having a bad reputation in a population of CDCC players is that one fails to receive cooperation in a public situation, which costs him pb per round. Therefore cooperation pays when meeting with a recipient in a private interaction if $c < qpb/\bar{q}$, yielding the desired condition.

An intuition behind the ESS condition of γ =CDCD (honest), $b/c > \bar{q}/q$, is described in the main text.

5 Numerical Calculations

5.1 Methods

To confirm our analytical predictions above, we have performed numerical calculations. The argument below is based on an infinitely large population, where players interact randomly according to the assumption described in Section 1. Given ecological parameters $b, c, p_1, p_2, q_1, q_2, e_1, e_2$ and ω, and given a social norm, n, we study the competition among the sixteen action rules.

First, we number the sixteen action rules in a lexicographical order, as CCCC ($j = 1$), CCCD ($j = 2$), ..., and DDDD ($j = 16$). Let x^j be the frequency of j-th ($j = 1, \cdots, 16$) action rule in the population. Also let $g^j(t)$ denote the fraction of good players among j-th action rule players. A similar calculation to eq.(2.3) leads to

$$\begin{aligned}\frac{\mathrm{d}g^j}{\mathrm{d}t} &= \omega p_1 q_1 \{gF^j_{1,\mathrm{G}} + (1-g)F^j_{1,\mathrm{B}}\} + \omega p_2 q_2 \{gF^j_{2,\mathrm{G}} + (1-g)F^j_{2,\mathrm{B}}\} \\ &\qquad + \omega(1 - p_1 q_1 - p_2 q_2)g^j + (1-\omega)g - g^j \\ &= \omega\left[p_1 q_1 \{gF^j_{1,\mathrm{G}} + (1-g)F^j_{1,\mathrm{B}}\} + p_2 q_2 \{gF^j_{2,\mathrm{G}} + (1-g)F^j_{2,\mathrm{B}}\} - (p_1 q_1 + p_2 q_2)g^j\right] \\ &\qquad + (1-\omega)(g - g^j)\end{aligned} \tag{5.5}$$

(we omitted t), where $F^j_{i,\mathrm{G}}$ and $F^j_{i,\mathrm{B}}$ respectively represent the probability with which a j-th action rule player receives a good reputation when interacting with a good/bad recipient in a public($i = 1$)/private($i = 2$) interaction. The fraction of good players in the population is given by $g(t) = x^1 g^1(t) + \cdots + x^{16} g^{16}(t)$. For given frequencies of the action rules $(x^1, \cdots, x^{16})$, the simultaneous

solution to $\mathrm{d}g^j/\mathrm{d}t = 0$ ($j = 1, \cdots, 16$) gives us the equilibrium fraction of good players among j-th action rule players, which we will write as g^{j*} ($j = 1, \cdots, 16$). To numerically solve the equilibrium values we have used *gsl_linalg.h* in GNU Scientific Library.

Then we calculate payoff of each action rule. The expected payoff of a j-th action rule player per interaction is given by

$$\begin{aligned} W^j = & \frac{1}{2} \underbrace{\left[p_1 g^* \delta^j_{1,\mathrm{G}} + p_1(1-g^*)\delta^j_{1,\mathrm{B}} + p_2 g^* \delta^j_{2,\mathrm{G}} + p_2(1-g^*)\delta^j_{2,\mathrm{B}}\right] \cdot (-c)}_{\text{as a donor}} \\ & + \frac{1}{2} \underbrace{\left[p_1 g^{j*} x^{(\mathrm{I})} + p_1(1-g^{j*})x^{(\mathrm{II})} + p_2 g^{j*} x^{(\mathrm{III})} + p_2(1-g^{j*})x^{(\mathrm{IV})}\right] \cdot b}_{\text{as a recipient}}, \end{aligned} \tag{5.6}$$

where $\delta^j_{i,\mathrm{G}}$ and $\delta^j_{i,\mathrm{B}}$ respectively represent indicator variables (yes = 1, no = 0) that describe whether j-th action rule prescribes cooperation when meeting a good(G)/bad(B) player in a public($i = 1$)/private($i = 2$) interaction. Also, $x^{(\mathrm{I})}, x^{(\mathrm{II})}, x^{(\mathrm{III})}$ and $x^{(\mathrm{IV})}$ respectively represent the frequencies of players whose 1st (=meeting a good player in a public interaction), 2nd (=meeting a bad player in a public interaction), 3rd (=meeting a good player in a private interaction) or 4th (=meeting a bad player in a private interaction), digit of the action rule is cooperation. They are given by

$$\begin{aligned} x^{(\mathrm{I})} &= \underbrace{x^1}_{\mathrm{CCCC}} + \underbrace{x^2}_{\mathrm{CCCD}} + \underbrace{x^3}_{\mathrm{CCDC}} + \underbrace{x^4}_{\mathrm{CCDD}} + \underbrace{x^5}_{\mathrm{CDCC}} + \underbrace{x^6}_{\mathrm{CDCD}} + \underbrace{x^7}_{\mathrm{CDDC}} + \underbrace{x^8}_{\mathrm{CDDD}} \\ x^{(\mathrm{II})} &= \underbrace{x^1}_{\mathrm{CCCC}} + \underbrace{x^2}_{\mathrm{CCCD}} + \underbrace{x^3}_{\mathrm{CCDC}} + \underbrace{x^4}_{\mathrm{CCDD}} + \underbrace{x^9}_{\mathrm{DCCC}} + \underbrace{x^{10}}_{\mathrm{DCCD}} + \underbrace{x^{11}}_{\mathrm{DCDC}} + \underbrace{x^{12}}_{\mathrm{DCDD}} \\ x^{(\mathrm{III})} &= \underbrace{x^1}_{\mathrm{CCCC}} + \underbrace{x^2}_{\mathrm{CCCD}} + \underbrace{x^5}_{\mathrm{CDCC}} + \underbrace{x^6}_{\mathrm{CDCD}} + \underbrace{x^9}_{\mathrm{DCCC}} + \underbrace{x^{10}}_{\mathrm{DCCD}} + \underbrace{x^{13}}_{\mathrm{DDCC}} + \underbrace{x^{14}}_{\mathrm{DDCD}} \\ x^{(\mathrm{IV})} &= \underbrace{x^1}_{\mathrm{CCCC}} + \underbrace{x^3}_{\mathrm{CCDC}} + \underbrace{x^5}_{\mathrm{CDCC}} + \underbrace{x^7}_{\mathrm{CDDC}} + \underbrace{x^9}_{\mathrm{DCCC}} + \underbrace{x^{11}}_{\mathrm{DCDC}} + \underbrace{x^{13}}_{\mathrm{DDCC}} + \underbrace{x^{15}}_{\mathrm{DDDC}} . \end{aligned} \tag{5.7}$$

An intuition behind eq.(5.6) is as follows. The term inside the first square brackets represents the probability that a donor with j-th action rule cooperates with a random recipient. The one inside the second square brackets represents the probability that a random donor cooperates with a recipient with j-th action rule. Factors 1/2 reflect the fact that every player has equal chances of being a donor or recipient.

Given payoff values, we can study evolutionary dynamics of the sixteen action rules. Here we assume the separation of two time scales; that reputation (=the fraction of good players for each action rule) equilibrates much quicker than evolutionary changes of frequencies of action rules, $(x^1, \cdots, x^{16})$.

The former time scale is measured by time t as in eq.(5.5), whereas we will measure the latter time scale by τ. Then the change of frequencies of action rules is described by the replicator equation [88-90]:

$$\frac{\mathrm{d}x^j(\tau)}{\mathrm{d}\tau} = x^j(W^j - W), \tag{5.8}$$

where $W = x^1W^1 + \cdots x^{16}W^{16}$ is the average payoff in the population.

The procedure of our numerical calculation is as follows. First we set initial frequencies of the action rules. Then we compute equilibrium fraction of good players for each action rule, and compute its expected payoff. Those payoff values are used in the replicator equation, eq.(5.8), for a sufficiently small interval of time, $\Delta\tau$, to yield new frequencies of the action rules after time $\Delta\tau$. These new frequencies are then used as a new initial condition, and the process is repeated.

5.2 Results

We have performed numerical simulations for three different social norms in order to confirm our analytical predictions. The results are shown in Figure 4 in the main text and Figures A and B in this Supporting Information. Throughout the analysis we have used the following parameter values; $b/c = 5, (p_1, p_2) = (p, 1-p), (q_1, q_2) = (1, q), e_1 = e_2 = 0.03$ and $\omega \to 1$.

As we mentioned in the legend of Figure 4 in the main text, the top left panel shows the social norm that is studied. The top right panel in each figure shows parameter regions where a specific action rule becomes an ESS. Panels in the middle row with red-blue mosaic shows the result of pairwise invasion analysis. For each possible pair of action rules we checked if a 'wildtype' action rule (initial abundance = 0.99) is invaded by a 'mutant' action rule (initial abundance = 0.01). Invasion is deemed successful if the abundance of mutant action rules exceed 0.01 after a long run, in which case the corresponding cell is shown in red. Otherwise the cell is in blue. Therefore rows with only blue cells correspond to ESS action rules in our numerical analysis. Action rules that are analytically predicted to be ESS are shown in white characters with a blue background. Panels in the bottom row with yellow-black mosaic shows the result of the replicator dynamics analysis. An 'initially abundant' action rule has the initial abundance of 0.99, whereas all the other action rules have the initial abundance of 0.01/15. In the 'all equal' condition, the initial abundance of all the sixteen action rules are set equal to 1/16. Abundance of each action rule after a long run is shown

by using a yellow-black scale. If a diagonal cell (borders highlighted in orange) is in full yellow, it implies that the action rule of the corresponding row is shown to be ESS in our numerical analysis. Action rules that are analytically predicted to be ESS are shown in yellow characters with a black background.

In Figure A, we studied the social norm under which cooperation is always deemed good but defection is deemed good only if it is done against a bad recipient in a public interaction (classified as ESS 3β and 3ϵ in Section 3.3). We studied three different combinations of (p, q), I:$(0.25, 0.25)$, II:$(0.1, 0.8)$, and III:$(0.8, 0.8)$. Pairwise invasion plots (assuming two action rules present) as well as replicator dynamics analysis (assuming all the sixteen action rules present) confirm that they are in agreement with our analytical predictions.

In Figure B, we studied the social norm under which cooperation is always deemed good but defection is deemed good only if it is done against a bad recipient in a public interaction or done against a good recipient in a private interaction (classified as ESS 9δ and 9ϵ in Section 3.3). We studied four different combinations of (p, q), I:$(0.25, 0.25)$, II:$(0.1, 0.8)$, III:$(0.4, 0.8)$, and IV:$(0.8, 0.8)$. Again numerical calculations are in agreement with our analytical predictions. Note that in the region containing the parameter III, we analytically predict mutual invasibility between CDDC and CDDD; either one can invade the other.

References in Supporting Information

88. Taylor PD, Jonker LB. Evolutionary stable strategies and game dynamics. Math Biosci. 1978; 40(1-2):145-156.

89. Hofbauer J, Sigmund K. Evolutionary games and population dynamics. Cambridge: Cambridge University Press; 1998.

90. Nowak MA. Evolutionary dynamics. Cambridge MA: Harvard University Press; 2006.

Figure Legends of Supporting Information

Figure A We numerically studied the evolutionary dynamics of action rules under the social norm presented in the top left table, where defection against a good individual in a public interaction and defection in a private interaction are deemed bad. For three different combinations of (p, q), I:$(0.25, 0.25)$, II:$(0.1, 0.8)$, and III:$(0.8, 0.8)$ (top right), we performed pairwise invasion analysis (panels in red & blue in the middle row) and replicator dynamics analysis (panels in yellow & black in the bottom row). In the invasion analysis, blue represents that the row action rule is robust against the invasion attempt by the column action rule, otherwise it is shown in red. In the replicator analysis, yellowness represents the frequency of action rules in the long run. Action rules that are predicted to be evolutionarily stable by our analytical calculations are highlighted with a different background color. See also the main text.

Figure B Numerical calculations of the evolutionary dynamics of action rules under the social norm presented in the top left table, where defection against a good individual in a public interaction and defection against a bad individual in a private interaction are deemed bad. For four different combinations of (p, q), I:$(0.25, 0.25)$, II:$(0.1, 0.8)$, III:$(0.4, 0.8)$, and IV:$(0.8, 0.8)$ (top right), we performed pairwise invasion analysis (panels in red & blue in the middle row) and replicator dynamics analysis (panels in yellow & black in the bottom row). Notation is the same as in Figure A.

Figure A

	Social Norm			
	Public		Private	
	Good	Bad	Good	Bad
C	**G**	**G**	**G**	**G**
D	**B**	**G**	**B**	**B**

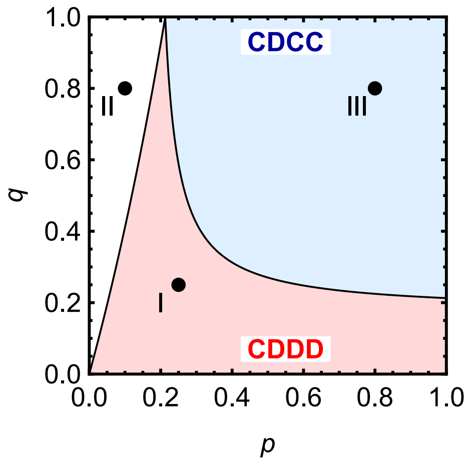

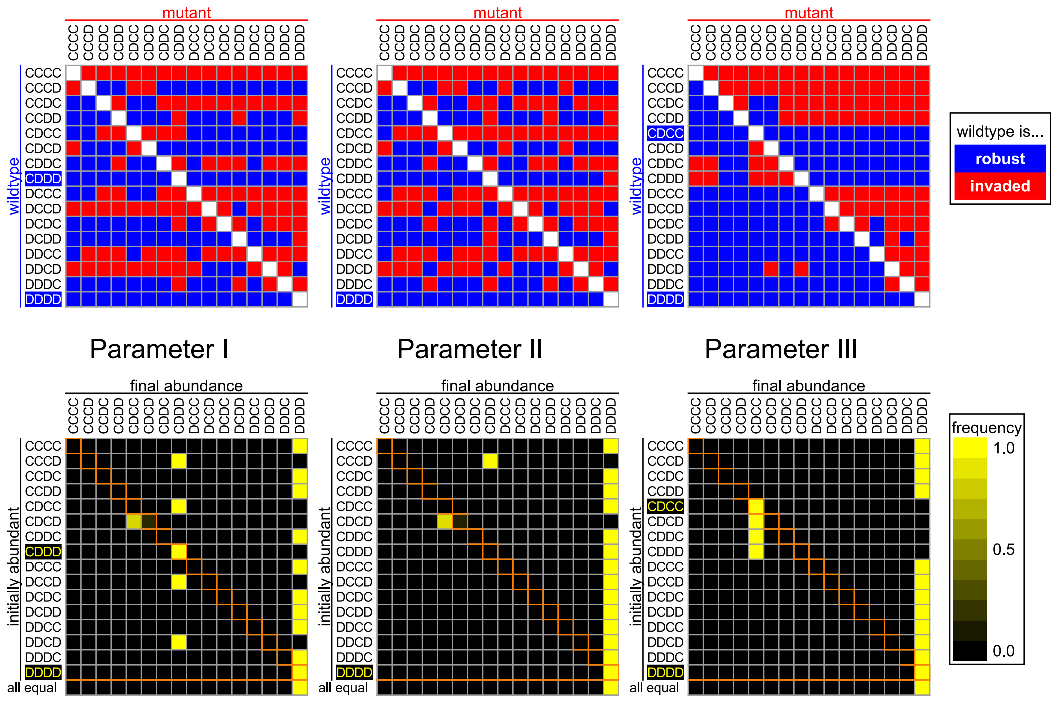

Figure B

	Social Norm			
	Public		Private	
	Good	Bad	Good	Bad
C	**G**	**G**	**G**	**G**
D	**B**	**G**	**G**	**B**

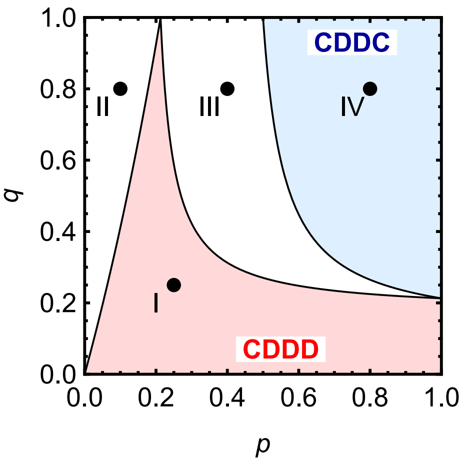

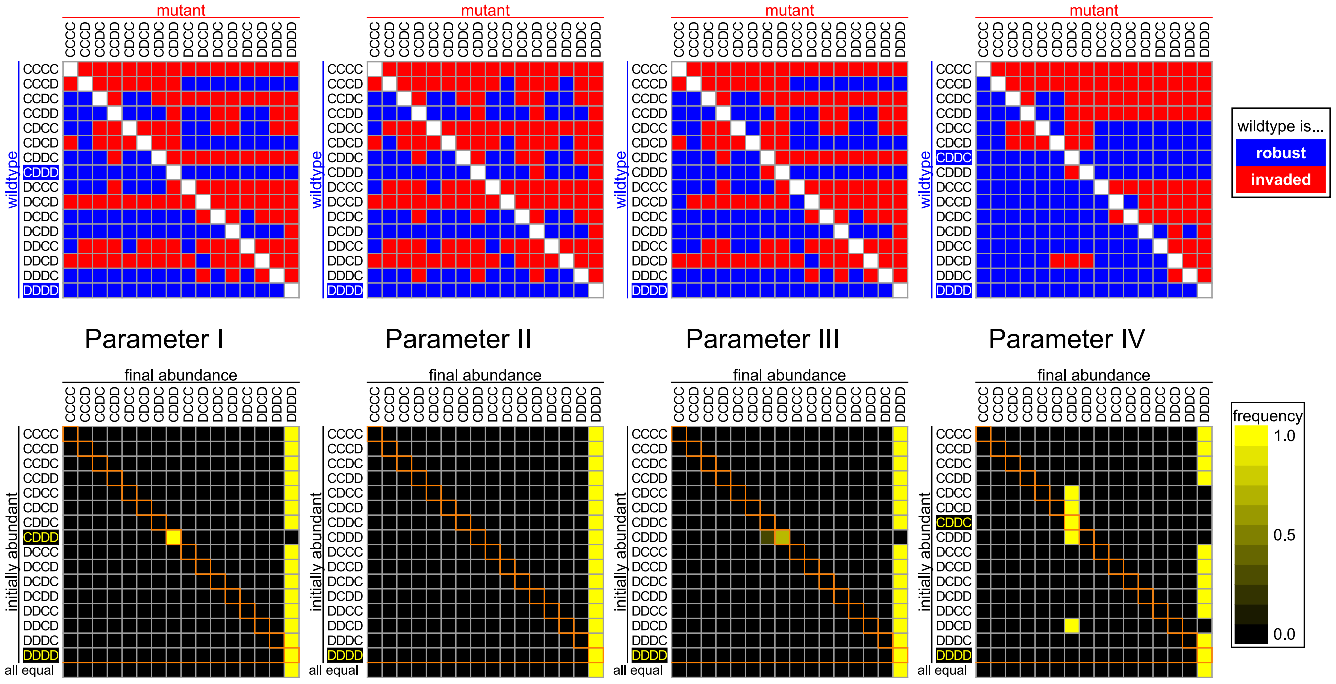

Supplement: S1 Text — The supporting information is structured as follows. Section 1 describes our full model with maximal generality. Section 2 describes the analytical framework to analyze this full model. Section 3 shows the results of our ESS search. Section 4 explains a reduced model, which directly corresponds to the model described in the main text. Section 5 describes numerical simulations. (PDF) [file pcbi.1004527.s001.pdf]
